# Clonal structure through space and time: High stability in the holothurian *Stichopus chloronotus* (Echinodermata)

**DOI:** 10.1002/ece3.3285

**Published:** 2017-08-14

**Authors:** Agathe Pirog, Pauline Gélin, Alexandre Bédier, Grégoire Bianchetti, Stéphane Georget, Patrick Frouin, Hélène Magalon

**Affiliations:** ^1^ UMR ENTROPIE (Université de La Réunion, IRD, CNRS) Saint Denis, La Réunion France; ^2^ Laboratory of Excellence CORAIL Perpignan France

**Keywords:** microsatellite, multilocus genotype, network, sea cucumber, *Stichopus chloronotus*

## Abstract

Sea cucumbers are increasingly exploited for human consumption and for their curative properties, and many wild populations are now depleted or in danger of extinction. While aquaculture is seen as an alternative to fisheries and as a mean to restore wild populations, more knowledge is needed on their reproductive strategies to render this practice efficient, notably for fissiparous holothurians, which are some of the mobile animals able of asexual reproduction by transverse fission. Little information is available on their population genetic diversity and structure. Here, the clonal structure of populations of the fissiparous sea cucumber *Stichopus chloronotus* has been investigated using nine microsatellite loci and a random sampling, at different spatial (intra‐reef and inter‐reef) and temporal (inter‐season and inter‐year) scales. Our findings highlight the importance of asexual reproduction in maintaining these populations, and the prevalence of the “initial seedling recruitment” strategy (ISR), leading to a high stability of clonal composition over seasons and years. It also seemed that clonal propagation was limited to the reef scale (<10 km) while reefs were connected by sexual dispersal. This is the first time that clonal structure in sea cucumbers has been studied at such a fine scale, with a specific sampling strategy. It provides key findings on the genetic diversity and structure of fissiparous sea cucumbers, which will be useful for the management of wild populations and aquaculture.

## 1 INTRODUCTION

Studying and understanding the way species reproduce (sexually, asexually, or both) are not only an academic matter, but are of practical importance for species management because of the consequences on population genetic diversity, connectivity, and dynamics. While sexual reproduction allows to maintain genetic diversity, asexual reproduction leads to the rapid propagation of the fittest genotypes and their predominance in the populations (Hamilton, 1980; Williams, 1975). Assessment of sexual and asexual reproduction in the wild thus allows to predict population ability (1) to adapt to disturbances (thanks to a sufficient genetic diversity) (Felsenstein, 1974; Waxman & Peck, 1999) and (2) to colonize rapidly new environments and maintain locally (thanks to asexual reproduction). Thus, species reproducing both sexually and asexually may have a considerable potential to evolve because they receive benefits from both modes. Nevertheless, these organisms generally favor one mode over the other, leading to strong implications for the evolution of populations and species: Different recruitment strategies (sexual or asexual) will affect dispersal (not performed by the same entities) and spatial occupation and will determine which entities (the alleles or the clonal lineages) will persist and evolve under natural selection (see Ayala, 1998). Besides the theoretical aspect, determining the modes of reproduction and their relative parts is of particular interest for organisms of economic interests. For example, in agronomy, asexual reproduction may lead to the rapid fixation of any desired genotype, while sexual reproduction will maintain a sufficient genetic diversity to sustain genetic improvement over the long term and to limit vulnerability to disease and pest epidemics (Hamilton, 1980; McKey, Elias, Pujol, & Duputié, 2010). Influence of the mode of reproduction has been poorly studied in animal breeding, as few of them reproduce asexually. Nevertheless, among exploited marine species, sea cucumbers (holothurians, Echinodermata) are good candidates.

Performing sediment bioturbation (Franklin, 1980; Purcell, 2010; Uthicke, 1999), holothurians provide a vital ecosystem service, notably for coral reefs, several species being described as “keystone species” (Paine, 1969; Power et al., 1996; Uthicke, Schaffelke, & Byrne, 2009). Meanwhile, presenting a high commercial value, approximately 66 species, mainly with a tropical distribution, are currently exploited by fisheries over the world (Conand, 2008; Purcell, 2010; Purcell, Hair, & Mills, 2012; Purcell, Samyn, & Conand, 2012). For the last thirty years, exploitation of wild populations has increased sevenfold and populations of the most exploited species (e.g., *Apostichopus japonicus*,* Holothuria scabra*,* Isostichopus fuscus*,* H. lessoni*,* H. nobilis*,* H. whitmaei*, and *Thelenota ananas*) become depleted and are now subjected to an extinction risk (Purcell, Polidoro, Hamel, Gamboa, & Mercier, 2014). Unsurprisingly, exploitation now turns toward less valuable species, such as *Stichopus chloronotus*,* S. hermani*, or *H. edulis* (Conand, 2008). Thus, to limit overfishing, aquaculture has become more and more developed, but needs more knowledge about (1) species modes of reproduction, (2) population genetic diversity to avoid genetic risk (i.e., introduction of new animals compromising the genetic integrity of local populations by large scale translocations and inbreeding), and (3) pathology.

Until now, only sexual holothurian species have been studied for aquaculture production, but their breeding remains difficult as they are broadcast spawners, leading to restricted successful fertilization and low larval survival rates (Eriksson, Robinson, Slater, & Troell, 2012). Besides, 16 holothurian species, which are exploited and could be bred in aquaculture, are able of asexual reproduction by transverse fission (reviewed in Dolmatov, 2014). Fission results into two new sections, and each regenerates the respective missing organs and tissues to form a complete animal, thus leading to clonal propagation. For most tropical fissiparous holothurians, sexual and asexual reproductions are asynchronous in time, occurring during the warm and the cold seasons, respectively (Conand, Jerome, Dijoux, & Garryer, 1998; Conand, Uthicke, & Hoareau, 2002; Uthicke, 1997), with some variations among species (Lee, Uthicke, & Byrne, 2009; Purwati, 2004). It has thus been hypothesized that fission in holothurians would maintain population stability, even when recruitment is low (Chao, Chen, & Alexander, 1994; Conand, 1996; Emson & Mladenov, 1987; Mladenov, 1996; Uthicke, 1997; Uthicke, Benzie, & Ballment, 1998). However, why these distinct reproductive strategies would occur at different rates remains unclear.

Asexual reproduction in holothurians has been particularly studied in *S. chloronotus*, the greenfish sea cucumber (Conand et al., 1998; Reichenbach & Holloway, 1995; Uthicke, Benzie, & Ballment, 1999; Uthicke & Conand, 2004; Uthicke, Conand, & Benzie, 2001). This species is found in the Indian and Pacific Oceans, inhabiting reef flats and back‐reef areas that are less than 20 m deep (Franklin, 1980). While presenting a moderate commercial value (Conand, 2008) but easily accessible for harvesting by hand or by free diving, it is exploited throughout its distribution range in artisanal and semi‐industrial fisheries, especially in Indonesia (Purcell, Hair, et al., 2012; Purcell, Samyn, et al., 2012). In the Western Indian Ocean, it is notably fished in Mauritius, Madagascar, and Kenya, for exportation to the Asian market (Conand et al., 2007; Muthiga & Conand, 2014; Purcell, Hair, et al., 2012; Purcell, Samyn, et al., 2012). Aquaculture production of this species is thus under consideration, but requires a better understanding of the relative contribution of sexual and asexual reproductions in the maintenance and the genetic diversity of its populations to be efficient. Sexual and asexual reproductions in this species occur generally during the warm and the cold seasons, respectively (Conand et al., 1998, 2002; Uthicke, 1997). High fission rates were observed in the Great Barrier Reef and Reunion Island (from 19% to 60% according to the population; Conand et al., 2002; Uthicke, 1997). The high prevalence of asexual reproduction in these populations has been confirmed using molecular markers (allozymes and then AFLP) by clone identification (Uthicke & Conand, 2004; Uthicke et al., 1999, 2001). Nevertheless, the contribution of asexual reproduction to the replenishment of local populations has only been assessed over short time frames and may vary seasonally and over time, as well as the occurrence of new genotypes through sexual reproduction.

Using nine microsatellite loci, we investigate the clonal diversity and composition in populations of the fissiparous holothurian *S. chloronotus* from Reunion Island (Western Indian Ocean), where fishing is forbidden and thus does not represent a selection pressure. This was done through space and time and at a finer scale than previously achieved, to assess the relative roles of asexual and sexual reproductions in the maintenance of these populations and their genetic diversity. Indeed, performing a random sampling scheme, we studied the genetic structure (1) at different spatial scales, from the intra‐reef to the inter‐reef, to estimate clonal dispersal distances, and (2) at different temporal scales, from the inter‐season (cold and warm seasons) to the inter‐year (two years apart), to study clonal persistence through time.

## 2 MATERIALS AND METHODS

### 2.1 Sampling

Sampling of the greenfish sea cucumber *S. chloronotus* was conducted at four reefs from the west coast of Reunion Island (21°06′S, 55°36′E), South Western Indian Ocean, 700 km east from Madagascar. Individuals were sampled one by one *in situ* in the back‐reef area (1–1.5 m depth), while snorkeling. For each individual, one or two papillae were instantaneously collected using cutting pliers (not destructive for the animal), and the longitudinal size was measured before releasing the animal where it was found. Samples were preserved in 95% ethanol and stored at room temperature.

#### 2.1.1 Spatial sampling

Six study sites were defined according to the density of *S. chloronotus*, in the back‐reef area of each reef. This species shows an aggregative distribution with a succession of high‐density patches (between 1 ind.m^−2^ and 3.15 ind.m^−2^; Conand et al., 1998, 2002) and zones of low densities (around 0.37 ind.m^−2^; Frouin P. personal communication), along the west coast of Reunion Island.

##### High‐density sites

**Figure 1:**
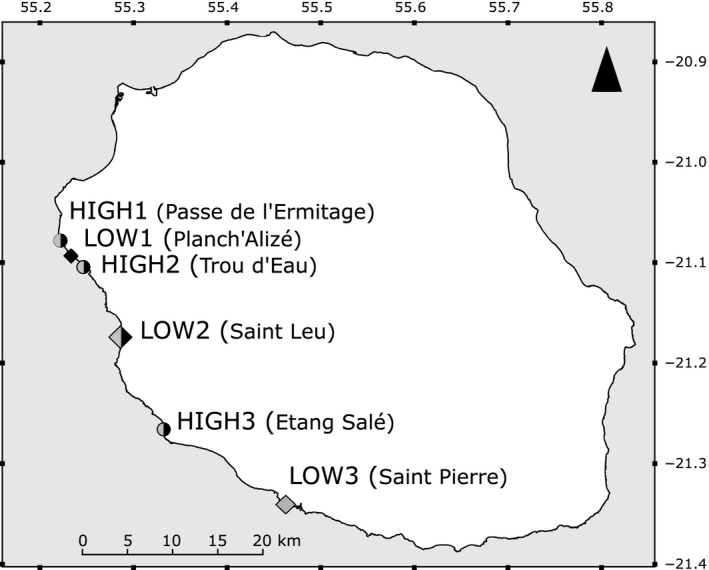
Map of the sampling sites of *Stichopus chloronotus* in Reunion Island. Circles represent high‐density sites while diamonds low‐density sites. In black, sites sampled during T0; in gray, sites sampled during T2

Three sites were chosen because of their high densities (named hereafter HIGH1, HIGH2, and HIGH3; Figure 1, Table 1), to study clonal structure at a fine scale. For each of these sites, we sampled two replicates, called stations (named S1 and S2), to assess clonal variations at the site scale. They were chosen near the center of the aggregation patch, approximately 150 m apart. For each of these stations, a random sampling procedure was performed in nested circular plots (radii of 5 m, 10 m, 20 m and 40 m) according to Baums, Miller, and Hellberg (2006). Within each circular strip, 16 individuals were randomly chosen. Thus, for each station, 64 individuals were sampled. Practically, GPS coordinates of the centers of each station were recorded and coordinates in each strip were generated randomly with increments set to 10 degrees and 10 cm, to avoid resampling of the same individual. For samples collected in circles of radii 5 m and 10 m, coordinates were located using a compass and a measuring tape attached to the center of the station. For samples collected in circles of radii 20 m and 40 m, coordinates were located using a GPS device. The nearest individual to each coordinate was sampled.

**Table 1:** Sampling of *Stichopus chloronotus* populations in Reunion Island for both sampling periods and for both seasons (cold + warm)

Reef	Locality	Sites	*N* T0 (2 stations)	*N* T2 (1 station)	Latitude (S)	Longitude (E)
La Saline‐Ermitage reef	Passe de l'Ermitage	HIGH1	128 + 128	64 + 64	21°05′5.58″	55°13′32.39″
La Saline‐Ermitage reef	Trou d'Eau	HIGH2	128 + 128	64 + 64	21°06′11.52″	55°14′44.61″
Étang Salé	Étang Salé	HIGH3	128 + 128	64 + 64	21°16′5.89″	55°19′57.29″
La Saline‐Ermitage reef	Planch'Alizé	LOW1	32 + 32		21°05′41.70″	55°14′1.65″
Saint Leu	Saint Leu	LOW2	32 + 32	48 + 48	21°10′41.76″	55°17′10.58″
Saint Pierre	Saint Pierre	LOW3		48 + 48	21°20′32.85″	55°27′41.44″
Total			896	576		

##### Low‐density sites

To study clonal propagation among *S. chloronotus* patches along the west coast of Reunion Island and to test the influence of population density on clonal structure, three other sites presenting low densities of *S. chloronotus* were added (named hereafter LOW1, LOW2, LOW3; Figure 1, Table 1). Individuals were haphazardly sampled over hundreds of meters while snorkeling parallel to the coast. For each site, 32 individuals were sampled.

##### Location of sampling sites in Reunion Island reefs

Among these sites, three (HIGH1, LOW1, and HIGH2) were located in the reef complex of La Saline‐Ermitage, approximately 7.5 km long. The three other sites (LOW2, HIGH3, and LOW3) were located in three distinct reefs: Saint Leu (2 km long), Étang Salé (1 km long), and Saint Pierre (2 km long), respectively (Figure 1, Table 1). HIGH1/LOW1/HIGH2 group in La Saline‐Ermitage complex and LOW2 were 9 km apart while LOW2 and HIGH3 were distant by 15 km and HIGH3 and LOW3 by 16 km (Figure 1).

#### 2.1.2 Temporal sampling

As the relative importance of each mode of reproduction may vary according to the season, sampling was performed in the cold season (October) and the successive warm season (February–March), and this two years apart, in 2013/2014 (T0) and 2015/2016 (T2).

##### T0

For T0, the exact same sampling scheme was performed for both seasons following the methodology of spatial sampling described above, but LOW3 was not sampled. A total of 896 individuals were thus sampled.

##### T2

For all high‐density sites, preliminary analyses performed before T2 did not highlight variations in the clonal structure between both stations within site (see Section 3). Thus, we sampled only one station for each site during T2. Furthermore, we decided not to sample once again at LOW1 as it did not show any differentiation with HIGH1 during T0, and we added another reef not sampled at T0, at the southern limit of the west coast of Reunion Island, showing low densities of *S. chloronotus* (LOW3) (Figure 1). Moreover, during T2, we sampled 48 individuals per low‐density site. In sum, during T0 and T2, two low‐density sites were sampled: LOW1/LOW2 (32 individuals per site) and LOW2/LOW3 (48 individuals per site), respectively. Thus, 576 individuals were sampled during T2.

#### 2.1.3 Density

During T2, for each high‐density site, population densities were measured using a 1 x 1 m quadrat, randomly thrown at each sampling point and all *S. chloronotus* individuals within the quadrat were counted. We thus performed 64 density measures for each high‐density site, for each season, during T2.

In total, 1,472 individuals were sampled during this study (Table 1).

### 2.2 Laboratory procedures

Total genomic DNA was extracted using Qiagen DNeasy Blood & Tissue kit (Qiagen, Hilden, Germany) from one papilla.

We tested microsatellite loci previously developed and characterized for *S. chloronotus* (10 loci; Taquet, Nagai, Yasuda, & Nadaoka, 2011), *S. horrens* (16 loci; Yuan, Xia, Zhang, & Hu, 2012), and *S. monotuberculatus* (15 loci; Xia, Hu, Fan, Luo, & Zhang, 2010). These 41 microsatellite loci were tested on eight individuals (six from Reunion Island and two from New Caledonia).

From the 41 microsatellite loci tested, 13 amplified and were easily readable and thus were selected to genotype all the samples: the 10 microsatellite loci developed for *S. chloronotus* (Taquet et al., 2011), as well as three developed for *S. monotuberculatus* (Sm007, Sm010, and Sm014; Xia et al., 2010). Among these 13 microsatellite loci, four were monomorphic overall our samples (Sc08, Sc20, Sc41, and Sm010) and we thus kept nine polymorphic loci to perform analyses on the 1,472 sampled individuals.

Genotyping was performed similarly for polymorphic tests and total genotyping. Forward primers were indirectly fluorochrome labeled (6‐FAM, VIC, NED) and were multiplexed post‐PCR in two panels (Appendix S1). Amplification reactions as well as the thermocycling program was similar as previously described in Postaire, Aurelle, Bourmaud, Bruggemann, and Magalon (2015). PCR products were genotyped using an ABI 3730 genetic analyzer (Applied Biosystems, Foster city, CA), and allelic sizes were determined using GeneMapper v 4.0 (Applied Biosystems, Foster city, CA).

### 2.3 Data analyses

#### 2.3.1 Analysis of clonal structure

##### Clonal diversity

To analyze the clonal structure, we only kept individuals whose multi‐locus genotypes (MLG) presented no missing data. First, the number of distinct MLGs (*N*_*MLG*_) was assessed, and then, the clones were identified, a clone being defined as a set of individuals that present the same MLG, using the package RClone (Arnaud‐Haond, Duarte, Alberto, & Serrao, 2007; Bailleul, Stoeckel, & Arnaud‐Haond, 2016). To assess the relevance of the loci used (number and variability), we estimated the probability that two individuals randomly sampled share exactly the same alleles over all loci (i.e., the same MLG) just by chance rather than the result of asexual reproduction (probability of identity *P*_*ID*_; Waits, Luikart, & Taberlet, 2001) with GIMLET v. 1.3.3 (Valière, 2002). Then, for identical MLGs, the probability *P*_*SEX*_(*F*_*IS*_) of detecting more than once the same MLG issued from distinct reproductive events was estimated using RClone. This probability takes into account possible departure from Hardy–Weinberg equilibrium (HWE) (Arnaud‐Haond & Belkhir, 2007).

To assess the relative part of asexual reproduction, the clonal richness *R* was calculated for each population (Dorken & Eckert, 2001), a population being defined by all the individuals sampled at the same place (station or site) and the same date. To discriminate identical MLGs due to asexual reproduction from those due to the potential weak variability of the loci used, clonal richness was assessed from simulated populations obtained without fission. These populations were composed of the individuals obtained at the next generation for each observed population, considering (1) only sexual reproduction including selfing (i.e., no fission), (2) panmixia, (3) unlinked loci, and (4) constant population size. Only T0 cold season (T0_cold_)was considered, as no significant clonal composition changes between seasons and sampling periods were observed (see Section 3). The simulated multilocus genotypes were created by randomly sampling two alleles for each of the studied loci in the gamete pool (corresponding to the allele frequencies in each observed population). For each observed population, 10^3^ datasets were simulated, and a mean clonal richness *R*_*sim*_ was then assessed, as well as the 95% confidence interval and the probability to observe a clonal richness weaker than the one observed (*P*‐value).

Furthermore, slightly different MLGs may be due to somatic mutations or scoring errors, corresponding to the same multi‐locus lineage (MLL) (Arnaud‐Haond et al., 2007), possibly overestimating the number of clones. Pairwise genetic distances between MLGs were computed, based on Rozenfeld's genetic distance (Rozenfeld et al., 2007) using RClone. From this distribution, a threshold was determined, under which genetic distances were considered to be due to somatic mutations or scoring errors, and characterized distinct MLGs belonging to the same MLL.

To assess clonal heterogeneity in the studied sites, the parameter *β* of the Pareto distribution and Pielou's evenness (*J*’) were estimated to describe whether the repeated MLGs were uniformly distributed rather than one predominant in each station.

The sampling design performed in the three high‐density sites allowed estimating the spatial arrangement of the clonemates. It was estimated using aggregation (*A*_*c*_) and edge effect (*E*_*e*_) indices, and their significance was tested with 10^3^ permutations using RClone.

##### Network analysis

A network was constructed using all distinct MLGs identified, to assess the genetic structure due to sexual reproduction at the island scale, whatever the season or the sampling period.

Then, to be able to compare the network indices between both sampling periods (T0 and T2) to assess the temporal variation in genetic structure, a network was also built for each period including all the distinct MLGs found at the common sites between both periods (HIGH1, HIGH2, LOW2, HIGH3). Finally, to assess the clonal structure at the site scale, a network was built for each station and for each season.

All networks were built using Rozenfeld's distance (Rozenfeld et al., 2007) with EDENetworks v. 2.18 (Kivelä, Arnaud‐Haond, & Saramäki, 2015). Nodes represent MLGs and links present the genetic distance between two MLGs. For each analysis, a “fully connected” network was built, including all links between all MLGs, and we then searched for the percolation threshold (Dpe) (Becheler, Benkara, Moalic, Hily, & Arnaud‐Haond, 2014; Moalic, Arnaud‐Haond, Perrin, Pearson, & Serrao, 2011; Rozenfeld et al., 2007), allowing to identify the first significant level of limitation of gene flow within the system, and thus different clusters (connected components). The clustering coefficient of the whole network < CC > was then assessed (Rozenfeld et al., 2007).

#### 2.3.2 Genetic diversity and differentiation

Asexual reproduction in *S. chloronotus* leads to two distinct individuals with the same MLG, and each can be considered to participate equally to the reproduction, either sexual (gamete broadcasting) or asexual. So, to perform genetic diversity and population structure analyses at the island scale, it seems relevant to keep all individuals, regardless of their MLG, to take into account the fact that a predominant MLG may have a better fitness than a rare one, even if it has been reported that considering all the members of a clone to assess differentiation indices may bias results. Noteworthy, when keeping one representative per MLG for each population (i.e., keeping only distinct MLGs), a very weak number of individuals remained to perform these analyses (Table 2; *N*_*MLG*_ ranging from 2 to 14 with a mean number of distinct MLGs per population of 6.03) and would induce too much bias.

**Table 2:** Indices of genetic diversity and clonal structure for *Stichopus chloronotus* populations from Reunion Island, at six different sites, at two seasons per year and two years apart: (a) T0: 2013/2014 and (b) T2: 2015/2016

(a)				T0											
Season	Reef	Site	Station	*N*	*N*_*MLG*_	*R*	*J*′	*β*	*A*_*c*_	*E*_*e*_	*N*_*a*_	*N*_*ap*_	*H*_*O*_	*H*_*E*_	*F*_*IS*_
Cold	La Saline‐Ermitage	HIGH1	S1	63	7	0.1	0.68	0.19	0.01	−0.17	2.00 (0.15)	0.00 (0.00)	0.49 (0.03)	0.28 (0.03)	−0.18***
			S2	64	7	0.1	0.61	0.14	−0.2	0.64	2.22 (0.20)	0.11 (0.04)	0.65 (0.02)	0.33 (0.03)	−0.32***
	La Saline‐Ermitage	HIGH2	S1	63	3	0.03	0.15	0.01	−0.01	0.39	1.56 (0.09)	0.00 (0.00)	0.75 (0.06)	0.17 (0.03)	−0.97***
			S2	64	6	0.08	0.22	0.02	0.17	0.22	2.11 (0.10)	0.44 (0.09)	0.44 (0.06)	0.18 (0.03)	−0.89***
	Etang Salé	HIGH3	S1	64	7	0.1	0.56	0.12	0.13	0.19	2.22 (0.10)	0.00 (0.00)	0.43 (0.05)	0.30 (0.03)	−0.29***
			S2	64	14	0.21	0.65	0.18	−0.13	0.1	2.33 (0.14)	0.00 (0.00)	0.35 (0.05)	0.27 (0.03)	−0.17***
	La Saline‐Ermitage	LOW1		31	5	0.13	0.78	0.24	‐	‐	1.89 (0.17)	0.00 (0.00)	0.61 (0.02)	0.31 (0.05)	−0.31***
	Saint Leu	LOW2		32	3	0.06	0.6	0.08	‐	‐	2.00 (0.18)	0.00 (0.00)	0.57 (0.07)	0.25 (0.04)	−0.54***
Warm	La Saline‐Ermitage	HIGH1	S1	62	8	0.11	0.62	0.16	−0.17	−0.16	2.00 (0.16)	0.00 (0.00)	0.43 (0.03)	0.25 (0.03)	−0.15***
			S2	64	7	0.1	0.72	0.2	0.09	0.24	2.00 (0.15)	0.00 (0.00)	0.48 (0.03)	0.26 (0.03)	−0.21***
	La Saline‐Ermitage	HIGH2	S1	64	4	0.05	0.17	0.01	0.15	−0.23	1.89 (0.12)	0.00 (0.00)	0.49 (0.07)	0.18 (0.03)	−0.87***
			S2	63	6	0.08	0.26	0.02	−0.22	0.38	1.89 (0.10)	0.00 (0.00)	0.51 (0.07)	0.18 (0.03)	−0.88***
	Etang Salé	HIGH3	S1	64	6	0.08	0.74	0.2	0.15	−0.78	1.89 (0.08)	0.00 (0.00)	0.44 (0.05)	0.26 (0.03)	−0.30***
			S2	64	7	0.1	0.57	0.11	−0.1	0.14	2.11 (0.10)	0.00 (0.00)	0.41 (0.05)	0.28 (0.03)	−0.29***
	La Saline‐Ermitage	LOW1		32	6	0.16	0.76	0.24	‐	‐	1.89 (0.16)	0.00 (0.00)	0.64 (0.02)	0.32 (0.05)	−0.33***
	Saint Leu	LOW2		31	5	0.13	0.62	0.13	‐	‐	1.78 (0.12)	0.00 (0.00)	0.56 (0.07)	0.26 (0.04)	−0.47***

(b)				T2											
Season	Reef	Site	Station	*N*	*N*_*MLG*_	*R*	*J*′	*β*	*A*_*c*_	*E*_*e*_	*N*_*a*_	*N*_*ap*_	*H*_*O*_	*H*_*E*_	*F*_*IS*_
Cold	La Saline‐Ermitage	HIGH1	S2	64	11	0.16	0.71	0.24	0.05	0.18	2.33 (0.20)	0.00 (0.00)	0.50 (0.02)	0.29 (0.03)	−0.14***
	La Saline‐Ermitage	HIGH2	S2	64	6	0.08	0.29	0.03	0.25*	0.39	2.11 (0.16)	0.11 (0.04)	0.48 (0.06)	0.19 (0.03)	−0.73***
	Etang Salé	HIGH3	S1	61	8	0.12	0.63	0.17	−0.02	0.44	2.33 (0.19)	0.11 (0.04)	0.52 (0.05)	0.31 (0.03)	−0.29***
	Saint Leu	LOW2		45	6	0.11	0.62	0.14	‐	‐	1.78 (0.10)	0.00 (0.00)	0.59 (0.05)	0.28 (0.03)	−0.43***
	Saint Pierre	LOW3		46	2	0.02	0.15	0.01	‐	‐	1.33 (0.07)	0.00 (0.00)	0.99 (0.00)	0.17 (0.04)	−0.99***
Warm	La Saline‐Ermitage	HIGH1	S2	63	7	0.1	0.64	0.15	0.07	−0.24	2.00 (0.15)	0.00 (0.00)	0.46 (0.03)	0.26 (0.03)	−0.20***
	La Saline‐Ermitage	HIGH2	S2	64	6	0.08	0.33	0.04	0.03	−0.18	1.78 (0.12)	0.00 (0.00)	0.58 (0.06)	0.20 (0.03)	−0.60***
	Etang Salé	HIGH3	S1	64	5	0.06	0.55	0.09	0.12	0.19	2.11 (0.15)	0.00 (0.00)	0.54 (0.05)	0.30 (0.03)	−0.42***
	Saint Leu	LOW2		48	3	0.04	0.84	0.26	‐	‐	1.78 (0.10)	0.00 (0.00)	0.64 (0.04)	0.29 (0.03)	−0.46***
	Saint Pierre	LOW3		48	2	0.02	0.15	0.01	‐	‐	1.44 (0.08)	0.00 (0.00)	0.76 (0.07)	0.17 (0.04)	−0.99***

*N*: number of individuals presenting no missing data; *N*_*MLG*_: number of distinct multilocus genotypes; *R*: clonal richness; *J′*: Pielou's index; *β*: parameter of the Pareto distribution (−1 * regression slope); *A*_*c*_: aggregation index; *E*_*e*_: edge effect. For *A*_*c*_ and *E*_*e*_ indices, none value was significantly different from 0, except one (*p < 0.05). *N*_*a*_: mean allelic number per site, *N*_*ap*_: mean number of private alleles per site, *H*_*O*_ and *H*_*E*_: observed and expected heterozygosities, respectively. In parentheses: standard errors. *F*_*IS*_: inbreeding coefficient and significant deviations from Hardy–Weinberg equilibrium are indicated as follows: *P < .05; **P < .01; ***P < .001.

Indices were assessed for each population. The mean number of alleles (*N*_*a*_) was assessed using FSTAT v. 2.9.3.2 (Goudet, 1995) and the mean number of private alleles per site (*N*_*ap*_) using the R‐package PopGenKit v. 1.0 (Paquette, 2012). We also assessed the expected and observed heterozygosities, respectively, *H*_*E*_ and *H*_*O*_, and the inbreeding coefficient *F*_*IS*_ to test for departures from HWE using Arlequin v. 3.5.1.3 (Excoffier & Lischer, 2010).

Population differentiation indices, *F*_*ST*_ (Weir & Cockerham, 1984) and *D*_*est*_ (Jost, 2008), were estimated between stations of a same site and among sites for a given season and sampling period (spatial differentiation). Additionally, they were assessed among seasons and sampling periods for a same site (temporal differentiation). They were also assessed between pairs of high‐density sites (all sampling dates pooled), keeping either all individuals or only one representative per MLG, to study connectivity among sites due to sexual reproduction. Indeed, the number of distinct MLGs in high‐density sites (from 16 to 22) was greater than in low‐density sites, although remaining weak for these calculations even if all sampling dates were pooled.

#### 2.3.3 Statistical analyses related to population density and individual size

Among high‐density sites, we tested whether the variable “density” during T2 was significantly dependent on factors “site” (three levels: HIGH1/HIGH2/HIGH3) and “season” (two levels: warm/cold) using a two‐way ANOVA.

We tested the effects of the factors “density” (two levels: low/high) and “season” (two levels: cold/warm) on the variable “individual size,” performing a two‐way ANOVA.

When necessary, pairwise Student's *t*‐tests were performed. All these analyses were performed using the statistical software R (R Development Core Team 2004).

## 3 RESULTS

### 3.1 MLG identification

Over the 1,472 individuals genotyped, 1,456 (98.9%) presented MLGs without missing data. The variability of the microsatellite loci used here resulted in a probability of identity *P*_*ID*_ of 2.356 × 10^−3^.

Over the 1,456 individuals with MLGs without missing data, a total of 74 distinct MLGs were detected, and among them, 31 were shared by several individuals. Over these 31 MLGs, seven appeared to be the result of different reproductive events (*P*_*SEX*_(*F*_*IS*_) > 0.05). Six of these seven MLGs were rare and were identified in 21 individuals (1.4% of the whole dataset). The last one (MLG27) was identified in LOW3 and was the most represented at this site (92/94 individuals). Furthermore, when departure from HWE was not taken into account, individuals presenting this MLG likely originated from a same reproductive event (*P*_*SEX*_ = 1.46 × 10^−37^).

For each site (only T0_cold_ considered), the observed clonal richness was significantly lower than the ones calculated from the simulated populations without fission (Appendix S2), even for HIGH2, which represented very few distinct MLGs and very weak clonal richness.

To take into account somatic mutations and eventual scoring errors, the threshold in the genetic distance distribution was set at one mutation step, as the first gap in the distribution of pairwise allelic differences was found between 0 and two mutation steps, as in Schnittler and Eusemann (2010) and Gitzendanner, Weekley, Germain‐Aubrey, Soltis, and Soltis (2012). As pairwise MLGs differed by at least two mutation steps, all distinct MLGs were considered as distinct MLLs. To avoid redundancy, we further kept the term MLG rather than MLL for the 74 distinct MLGs found among our sampled individuals.

### 3.2 Genetic diversity

The genetic diversity of the loci used was low, with a number of alleles per locus varying from 2 (Sc01, Sc10, and Sm007) to 9 (Sc33) and the mean allele number per site (*N*_*a*_) from 1.33 to 2.33 (Table 2a,b). Private alleles [mean ± standard error (*SE*)] were identified at (T0cold) in HIGH1 and HIGH2 (*N*_*ap*_ = 0.11 ± 0.04 and 0.44 ± 0.09, respectively) and at T2_cold_ in HIGH2 and HIGH3 (*N*_*ap*_ = 0.11 ± 0.04).

*H*_*O*_ varied from 0.35 to 0.99 while *H*_*E*_ varied from 0.17 to 0.33 (Table 2a,b). Significant deviations from HWE were found for all populations, highlighting high heterozygote excesses (*F*_*IS*_ = [−0.99, −0.14]; *P* < .001; Table 2a,b).

### 3.3 Spatial distribution of clones

#### 3.3.1 Between stations within site

The sampling of two stations for each high‐density site (HIGH1, HIGH2, and HIGH3) during T0 allowed studying the clonal distribution at the intra‐reef scale. During T0_cold_, over the 384 individuals sampled in the three high‐density sites, 381 individuals presented MLGs without missing data and 32 distinct MLGs were identified. Among these MLGs, 15 were represented by several individuals and 17 were found only once.

**Figure 2:**
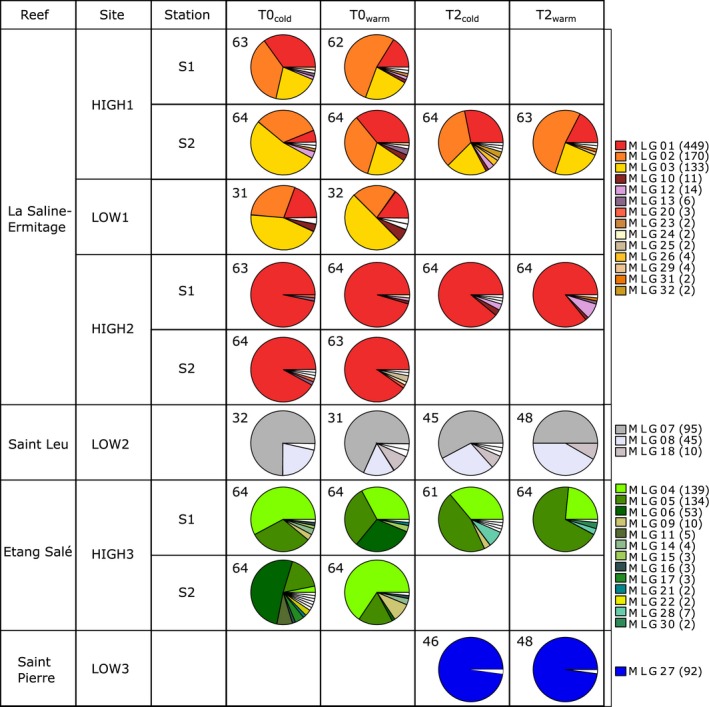
Clonal distribution within each station and each site for each sampling year and season. Each sector represents a multi‐locus genotype (MLG) and is proportional to the number of individuals presenting this MLG. A specific color was assigned to each MLG shared by at least two individuals, while MLGs presented by only one individual were left blank. Numbers in each cell indicate the number of individuals whose MLGs present no missing data. Numbers in parentheses in the legend indicate the number of individuals presenting the MLG

For a given high‐density site, both stations presented similar values for all the clonal parameters investigated here (*R*,* J*′, and *β*; Table 2a). All these values were weak indicating that each station was composed by one to three MLGs that were represented by most individuals, along with some rare ones (represented by one or two individuals). Above all, the most frequent MLGs were the same between stations: MLG01, MLG02, and MLG03 for HIGH1; MLG03 for HIGH2; MLG04, MLG05, and MLG06 for HIGH3. Some rare MLGs were also shared between both stations of each site, for instance MLG12 for HIGH1 (Figure 2). Whichever the station, no significant spatial aggregation of clonemates (*A*_*c*_, all *P *> .05) or significant edge effects (*E*_*e*_, all *P *> .05) were detected (Table 2a).

Finally, for all high‐density sites, even if some were significantly different from 0 due to repeated MLGs, low values of differentiation indices were found between stations from a same site (*F*_*ST*_ = [0.00, 0.04], *D*_*est*_ = [0.00, 0.02]; Table 3). Thus, within an aggregation patch (site), clonemates and individuals with unique MLGs were randomly distributed. Therefore, sampling at one station should allow characterizing asexual reproduction within the site.

**Table 3:** Genetic differentiation between stations sampled at T0_cold_ estimated with Weir and Cockerham's *F*_*ST*_ (lower‐left matrix) and Jost's *D*_*est*_ (upper‐right matrix) estimates

	HIGH1 S1	HIGH1 S2	HIGH2 S1	HIGH2 S2	HIGH3 S1	HIGH3 S2	LOW1	LOW2
HIGH1 S1	‐	0.02	0.07	0.06	0.06	0.07	0.00	0.13
HIGH1 S2	0.03	‐	0.13	0.12	0.10	0.11	0.00	0.14
HIGH2 S1	0.13	0.22	‐	0	0.03	0.02	0.09	0.20
HIGH2 S2	0.13	0.21	0.00	‐	0.03	0.02	0.09	0.20
HIGH3 S1	0.09	0.13	0.08	0.08	‐	0.01	0.08	0.21
HIGH3 S2	0.11	0.16	0.05	0.05	0.04	‐	0.09	0.19
LOW1	0.00	0.00	0.19	0.18	0.10	0.13	‐	0.13
LOW2	0.19	0.19	0.36	0.35	0.29	0.27	0.18	‐

white: *P *> .05; light gray: *P *< .01; gray: *P *< .001.

#### 3.3.2 Among sites

At T0_cold_, considering all the sites (i.e., 444 individuals with MLGs without missing data), 91.9% of the individuals belonged to one of the ten most represented MLGs and one MLG was over‐represented (MLG01: 34.2%). Interestingly, some of these MLGs were shared between HIGH1 and LOW1 (MLG01, MLG02, and MLG03) and among HIGH1, LOW1, and HIGH2 (MLG01), sites that belong to the same reef (3 km apart). Conversely, no MLGs were shared among sites located in different reefs, that is, among the complex HIGH1/LOW1/HIGH2, LOW2, and HIGH3 (Figure 2).

Indices of clonal richness *R* were very low for all sites, from 0.03 to 0.21 (Table 2a). All the sites presented similar values of Pielou's indices *J*′ (0.56–0.78), except HIGH2 that presented the lowest ones (0.15–0.22) (Table 2a). The parameter *β* of the Pareto distribution showed similar results: a very low value for HIGH2 (from 0.01 to 0.02), and higher values for the other sites, from 0.12 to 0.24 (Table 2a). Indeed, MLG01 was highly represented in HIGH2, from 59 to 61 individuals according to the station, representing 92%–97% of the individuals (Figure 2). None of these indices were significantly different between high‐density and low‐density sites (*R*: pairwise Wilcoxon test, *W* = 4, *P *= 1.00; *J*′: pairwise Wilcoxon test, *W* = 3, *P *= .43; *β*: pairwise Wilcoxon test, *W* = 4, *P *= 0.64).

Furthermore, differentiation indices were calculated among sites, pooling both stations for each site. They were highly significantly positive among all sites (*F*_*ST*_ = [0.06, 0.37] and *D*_*est*_ = [0.02, 0.20]; all *P *< 0.001) except between HIGH1 and LOW1 that belong to the same reef complex (Appendix S3a). Thus, as similar MLGs and no differentiation were found between these sites, LOW1 was not sampled at T2, but replaced by another low‐density site (LOW3), where two new MLGs were identified with one over‐represented (47/48 individuals).

**Figure 3:**
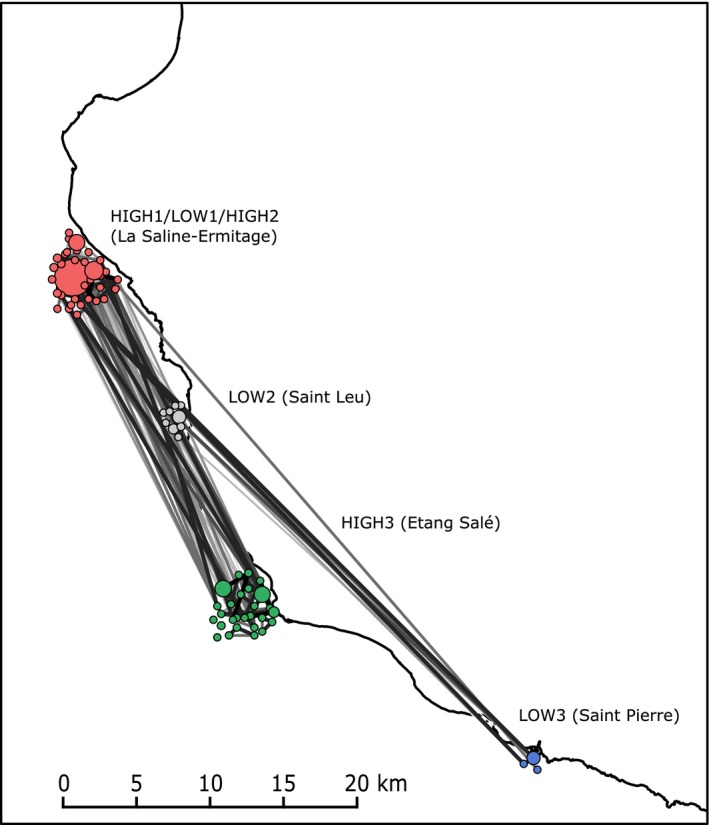
Network topology of multi‐locus genotypes (MLGs) identified in *Stichopus chloronotus* whole sampling based on the Rozenfeld's distance. Only links with distances smaller or equal to the percolation threshold (Dpe = 0.89) are presented. Nodes, representing MLGs, are arranged according to their geographic coordinates. Node size is proportional to the number of individuals harboring each MLG. Node colors correspond to reefs

Finally, the network constructed using all MLGs identified in the study (*N*_*MLG*_ = 74) did not highlight a clear geographic structuring, with strong links among MLGs sampled in different reefs (Figure 3). Furthermore, *F*_*ST*_ and *D*_*est*_ estimates calculated among high‐density sites using one representative per MLG (all sampling dates pooled) were lower (*F*_*ST*_ = [0.03, 0.04], all *P *< .01; *D*_*est*_ = [0.01, 0.03], all *P *< .05 except one) than those calculated keeping all individuals (*F*_*ST*_ = [0.06, 0.17] and *D*_*est*_ = [0.02, 0.09]; all *P *< .001) (Appendix S3b,c).

### 3.4 Temporal variation of clonal structure

#### 3.4.1 Interseasonal variability

For both sampling periods, similar dominant MLGs were found at each site for both seasons, but some rare MLGs appeared or disappeared between seasons (Figure 2). Mean clonal richnesses (*R*) were not significantly different between seasons for both sampling periods (T0: pairwise Wilcoxon test, *W* = 8, *P *= .42; T2: pairwise Wilcoxon test, *W* = 19, *P *= .22). Similar results were found concerning mean Pielou's indices *J*′ (T0: pairwise Wilcoxon test, *W* = 8, *P *= .42; T2: pairwise Wilcoxon test, *W* = 12, *P *= 1) and the parameter *β* of the Pareto distribution (T0: pairwise Wilcoxon test, *W* = 8, *P *= .42; T2: pairwise Wilcoxon test, *W* = 13, *P *= 1).

Differentiation indices were also estimated between both seasons at a given site, to test for temporal differentiation within sites. Low differentiation values were found for all sites between both seasons whatever the sampling period (*F*_*ST*_ = [0.000, 0.020]; *D*_*est*_ = [0.000, 0.011]; Table 4).

**Table 4:** Genetic differentiation between both seasons of a given sampling period (T0 or T2), and between sampling periods, estimated with Weir and Cockerham's *F*_*ST*_ (upper value) and Jost's *D*_*est*_ (lower value)

	T0 cold/warm	T2 cold/warm	T0/T2
HIGH1	0.012**	0.009	0.000
	0.007**	0.006	0.000
HIGH2	0.000	0.000	0.001**
	0.000	0.000	0.000
HIGH3	0.004	0.020*	0.047***
	0.002**	0.011**	0.027***
LOW1	0.000	‐	‐
	0.000	‐	‐
LOW2	0.000	0.000	0.013*
	0.000	0.000	0.006**
LOW3	‐	0.000	‐
	‐	0.000	‐

*P < 0.05; **P < 0.01; ***P < 0.001.

#### 3.4.2 Interannual variability

Among the 37 distinct MLGs identified during T2, 23 were new. Among these 23 MLGs, three were identified in LOW3 representing all the individuals of this site and the 20 others were rare, presented at most by seven individuals and representing 5.1% of the individuals sampled during T2 (Figure 2). These 20 new rare MLGs were not exclusively represented by small individuals (only 9.7% measuring less than 7 cm), which would have indicated that they were originated from the previous event of sexual reproduction. The 14 MLGs already found during T0 were the most frequent ones, representing 78% of the individuals sampled during T2. For each site, the most frequent MLGs at T2 were the same as in T0. Furthermore, in LOW2 and HIGH3, it seems that between the two sampling periods (T0_cold_ and T2_warm_), the frequency of the most represented MLG identified in each site in T0 decreased in favor of another one (MLG07 and MLG08 in LOW2; MLG04 and MLG05 in HIGH3; Figure 2). At a lesser extent, this pattern was also observed in HIGH2, with MLG12, that was not present at T0_cold_ but with a frequency of 7.8% at T2_warm_ (Figure 2).

Similar to T0, the clonal parameters were not significantly different between high‐density and low‐density sites (*R*: pairwise Wilcoxon test, *W* = 20, *P *= .11; *J*′: pairwise Wilcoxon test, *W* = 15, *P *= 0.61; *β*: pairwise Wilcoxon test, *W* = 15, *P *= 0.61). Likewise, differentiation indices were highly significantly positive among all sites (*F*_*ST*_ = [0.06, 0.37] and *D*_*est*_ = [0.02, 0.19]; all *P *< .001) (Appendix S3a). In order to compare clonal parameters between T0 and T2, LOW1 and LOW3 were not included in further analyses, as these sites were only sampled during one of the two periods and, for each high‐density site, only the station sampled at both periods was kept. We found no significant difference of clonal richness between both periods (pairwise Wilcoxon test, *W* = 31, *P *= 0.96), and similar results were obtained when studying the Pielou's index *J*′ (pairwise Wilcoxon test, *W* = 27, *P *= .65) and the parameter *β* of the Pareto distribution (pairwise Wilcoxon test, *W* = 23, *P *= .37).

Differentiation indices calculated for all sites between T0 and T2 were low, with *F*_*ST*_ values varying from 0.000 to 0.047 and *D*_*est*_ values varying from 0.000 to 0.027 (Table 4). Highest values were found in HIGH3 (*F*_*ST*_ = 0.047 and *D*_*est*_ = 0.027; *P *< .001) and may be related to the change in dominant MLGs frequencies.

The global network built on the basis of MLGs in T0 ($N_{{MLG}_{T0}}=29$) was rather similar to the one obtained in T2 ($N_{{\mathrm{MLG}}_{T0}}=34$) with identical percolation thresholds (Dpe = 1.11). The clustering coefficient < CC > was higher in T2 than in T0 (0.52 and 0.45, respectively), suggesting an increase in the hierarchical differentiation among clusters (Appendix S4).

In order to see whether this result was due to an increase in differentiation at the island or site scale, networks were also constructed for each station. Clustering coefficients were variable, with no clear pattern of variations (Appendix S5). Indeed, for one station, for example, HIGH1S2, the clustering coefficient increased between T0_cold_ and T0_warm_ (from 0.74 to 0.83) and decreased between T2_cold_ and T2_warm_ (from 0.45 to 0.31). It thus seems that the increase in the hierarchical differentiation among clusters highlighted at the island scale is not related to an increase at the site scale.

### 3.5 Variation in population density and individual size

#### 3.5.1 Population density

At T2, considering the high‐density sites only, the two‐way ANOVA showed that the population density was significantly dependent on the site (*F*(2,378) = 3.02; *P *< 10^−16^), but not on the season (*F*(1,378) = 3.87; *P *= .44) with no interaction between season and site (*F*(2,378) = 3.02; *P *= .17) (see Appendix S6b). Density (mean ± *SE*) was higher in HIGH2 (4.3 ± 0.3 ind.m^−2^), than in HIGH3 (2.3± 0.2 ind.m^−2^) and in HIGH1 (1.2 ± 0.1 ind.m^−2^) (pairwise Student's *t‐tests*; Appendix S6a,c).

#### 3.5.2 Individual size

The two‐way ANOVA showed that the size was significantly dependent on the season (*F*(1,1466) = 3.85; *P *< 2.2 × 10^−16^) and the density (*F*(1,1466) = 3.85; *P *= 2.56 × 10^−11^) with an interaction between both factors (*F*(1,1466) = 3.85; *P *= 3.12 × 10^−13^) (Appendix S6e). The size variation among high‐ and low‐density sites was more important in the warm season (mean ± *SE* = 12.3 ± 0.1 cm and 17.2 ± 0.4 cm, respectively) than in the cold season (mean ± *SE* = 11.7 ± 0.2 cm and 13.1 ± 0.4 cm, respectively) (Appendix S6d,f).

## 4 DISCUSSION

Our results revealed a high proportion of asexual reproduction in the greenfish sea cucumber *S. chloronotus* on the west coast of Reunion Island. Each population was composed of few clones, each with a lot of clonemates. At the island scale (six sites), *S. chloronotus* showed a weak clonal propagation among reefs while sexual dispersal seemed to be over longer distances. Additionally, sampling through time allowed detecting a high temporal stability in clonal composition, between seasons and years. Moreover, while the size of the individuals varied following the season and the density, this latter had no effect on clonal structure.

### 4.1 Importance of asexual reproduction in population maintenance

Using nine microsatellite loci, we detected a very low clonal richness at all sites, highlighting the importance of asexual reproduction in propagation and maintenance of *S. chloronotus* in Reunion Island. Nevertheless, we found a moderate value of *P*_*ID*_ (2 × 10^−3^) compared to other studies (10^−4^–10^−5^; Waits et al., 2001) and seven *P*_*SEX*_(*F*_*IS*_) that did not differ significantly from zero. This may indicate that a small proportion of the genotypic diversity may remain undetected, due to the lack of statistical power induced by datasets with a very low clonal diversity (Arnaud‐Haond et al., 2007). However, for the six *P*_*SEX*_(*F*_*IS*_) > 0.05 found for rare MLGs, even if these MLGs were the result of different reproductive events, the overestimation of asexual reproduction that it entailed might be very slight, because it implied a weak number of individuals compared to the whole dataset (1.4%). As for MLG27 (found in LOW3 and representing almost all the individuals sampled at this site), it is highly unlikely that most of the individuals presenting this MLG were not the result of fission. Nevertheless, we cannot rule out that some of these individuals might have been produced by sexual reproduction (notably between two individuals presenting this MLG). Furthermore, the simulated clonal richnesses considering sexual reproduction only were significantly greater than the ones observed, even for HIGH2 presenting very few MLGs and a very weak clonal richness. So the loci used are discriminant enough to retrieve most of the genetic diversity of the studied populations and to detect asexual reproduction.

The clonal richness was very weak for all the populations (0.02–0.21), indicating that asexual reproduction is preponderant in these populations. The importance of asexual reproduction in *S. chloronotus* has already been demonstrated studying fission rates. In the Great Barrier Reef, they varied from 19% to 43% according to the population (Uthicke, 1997) and, in Reunion Island, from 50% to 60% at Trou d'Eau (HIGH2 herein) and Etang Salé (HIGH3 herein), respectively (Conand et al., 2002). Then, it has been confirmed using allozymes (Uthicke et al., 1999) and AFLP (Uthicke & Conand, 2004). In *S. chloronotus* populations from Reunion Island, using allozymes, only one or two genotypes were retrieved per population (approximately 50 individuals) (Uthicke et al., 2001). Furthermore, using AFLP loci to study populations from the Western Indian Ocean (Reunion Island, Madagascar) and from the Great Barrier Reef, Uthicke and Conand (2004) detected 51 genotypes in the whole sample (*n* = 149), with up to 20 individuals (nearly all the individuals of a population) presenting the same genotype. Besides, the importance of asexual reproduction varied according to the population. One population in the Great Barrier Reef (Great Palm Island), one in Reunion Island (Trou d'Eau/HIGH2), and at a lesser extent in Saint Leu (LOW2 herein), exhibited higher levels of asexuality, with at least 79% of the sampled individuals belonging to the same clone (Uthicke et al., 1999). This pattern was confirmed here, with more than 90% of the individuals sampled in Trou d'Eau/HIGH2 presenting the same MLG. We also highlighted this pattern in Saint Pierre (LOW3 herein), site not studied previously and at the southern end of the island.

Five possible factors may influence fission rates: habitat instability (related to environmental stress), optimum individual size, food availability, juvenile mortality, and larval supply (Uthicke, 2001). Here, asexual reproduction does not seem to be related to individual size. Indeed, individuals were smaller in high‐density sites than in low‐density sites, especially at the warm season. However, no indices of clonal structure were significantly different between high‐ and low‐density sites, nor between seasons. Two sites showed an extreme clonal structure, HIGH2 and LOW3, with one MLG highly predominant (more than 90% of the individuals sampled). The importance of fission highlighted in HIGH2 (Trou d'Eau) may be related to higher food availability, as this site is subject to stream inputs enriched in nutrients (Cuet, Naim, Faure, & Conan, 1988; Naim, Cuet, & Mangar, 2000). This higher food availability may also explain the higher population density observed at this site, as already suggested (Conand et al., 1998, 2002; Uthicke et al., 2001). This high fission rate may be also due to a toxic stress induced by the input of pesticides and organic contaminants that are carried downstream into the reef. Kolasinski, Taddei, Cuet, and Frouin (2010) showed that *Holothuria leucospilota* individuals also sampled in HIGH2 (Trou d'Eau) presented either lower acetylcholinesterase (AChE) activity levels or faster Cuvierian tubules expulsion than in other sites of the La Saline‐Ermitage reef complex, which are two stress indicators. Nevertheless, this stress increase was not highlighted in sampled individuals of *H. atra* (Kolasinski et al., 2010) and may be species related. Likewise, in Saint Pierre (LOW3), freshwater inputs may explain the high fission rate observed. This reef is also submitted to currents and strong austral oceanic swell (Lecacheux et al., 2012). Thus, the high levels of asexual reproduction highlighted there may be related to high environmental instability. It would be interesting to test for stress indicators in *S. chloronotus* at all the study sites.

The predominance of one MLG in Saint Pierre (LOW3), and at a lesser extent in Saint Leu (LOW2), may also be explained by an important larval mortality due to specific environmental conditions. These sites also exhibited low densities of *S. chloronotus*, maybe due to a high post‐settlement mortality of larvae; the most frequent MLG found in each of these sites would then be the fittest to local environmental conditions. On the other hand, selection may not play any role, but just chance, as in gene surfing (Hallatschek, Hersen, Ramanathan, & Nelson, 2007) or equal chance hypothesis in ecology (Sale, 1979): the first to arrive, the first to propagate.

### 4.2 Clonal propagation in space

While echinoderms present various patterns of asexual reproduction, it only occurs by fission in holothurians (Dolmatov, 2014). One species only, *Parastichopus californicus*, has been found to reproduce asexually during the larval stage, with fission occurring at the doliolaria stage (Eaves & Palmer, 2003). For all other species investigated, fission only occurred at the adult stage. Thus, clonal dispersal in *S. chloronotus* may only be achieved by adults.

Movements of holothurians are difficult to assess, as tagging methods are not always well‐adapted, tags being rejected by the tegument according to the species (Conand, 1991, 1993). However, some studies managed to estimate the motion speed of *H. atra* and *H. leucospilota*, which was *ca*. 52 m.day^−1^ (Bakus, 1973) and 41 m.day^−1^ (Taddei, 2006), respectively. Thus, *S. chloronotus* may present similar motion speed. Here, shared MLGs were commonly found between both stations within site, which were separated by about 150 m. This pattern seems congruent with motion speeds highlighted in other sea cucumber species. Thus, *S. chloronotus* individuals may be highly mobile within an aggregation patch, at the scale of several hundred meters.

Furthermore, even if shared MLGs were found in sites located in the same reef, no common MLGs were identified among reefs, indicating that clonal propagation may be restricted to the reef scale. Using AFLP, Uthicke and Conand (2004) found two individuals in Trou d'Eau (HIGH2) presenting a genotype commonly identified in Saint Leu (LOW2), and one individual in the latter site that presented the genotype mainly identified in HIGH2. The fact that we did not identify shared MLGs among reefs may be related to the higher discriminant power of microsatellites. It may also be possible that the propagation of a MLG from one reef to another and its maintenance in the new reef is extremely rare.

Meanwhile, the lack of geographic structure in the networks built keeping one representative per MLG may represent gene flow among reefs due to sexual reproduction. This is confirmed by the lower *F*_*ST*_ and *D*_*est*_ estimates assessed among high‐density sites keeping one representative per MLG as compared to those assessed keeping all individuals, as well as the low or absent number of private alleles within each site. During sexual reproduction, gametes are released directly into the water column and fertilized eggs rapidly develop into free‐swimming planktotrophic larvae, the main dispersal stage of sea cucumbers. Little information on the larval life span is available, but larvae seem to spend a couple to several weeks in the water column before settling (reviewed in Smiley, McEuen, Chaffee, & Krishnan, 1991), possibly allowing dispersal among reefs. Thus, sexual reproduction may maintain connectivity among *S. chloronotus* patches of Reunion Island whereas clonal propagation may take place at the reef scale only.

### 4.3 Clone persistence through time

Sampling at both seasons within a year was performed because of higher fission rates in the cold season and a predominance of sexual reproduction in the warm season. Gamete spawning in this species occurs between November and February (Hoareau & Conand, 2002). Conand (1988) assessed the parameters of von Bertalanffy equation: $L_{t}=L_{\infty }(1-e^{-k(t-t_{0})}$), with *L*_∞_ = 342.24, *k* = 0.45, and *t*_0_ = −0.25, allowing to estimate the growth rate of *S. chloronotus*. Thus, when the warm season sampling occurred in March, the new individuals issued from sexual reproduction could be aged from 1 to 4 months, that is, measuring approximately from 4.7 to 7.9 cm long. Even if the mean size of the individuals sampled was higher (12.6 cm), 5%–20% of small individuals (< 8 cm) without fission evidence were sampled at each season for each period (90 individuals < 8 cm at T0_cold_, 70 at T0_warm_, 34 at T2_cold_, and 16 at T2_warm_). These small individuals should represent the genetic contribution of the sexual reproduction event from the previous season, remaining weak compared to the part of asexual reproduction. Furthermore, for each site studied two years apart, *S. chloronotus* patches were dominated by one to three MLGs, which were found in similar proportions, showing a high stability through time of their clonal composition, over several years. The networks built for each sampling period also presented a rather similar structure, confirming the stability of the populations’ genetic composition. Conversely, the majority of the rare MLGs (presented by few individuals, i.e., <5% for each population) were not retrieved from one sampling period to the other, highlighting the variability of these MLGs. This variability was also found in the frequency of certain MLGs, which decreased (MLG07 and MLG04) or increased (MLG08 and MLG05) between both sampling periods. Thus *S. chloronotus* populations from Reunion Island seem constituted of a core of large and stable clones, with some rare transient MLGs issued from sexual reproduction. The genotypic composition of these populations may be an indicator of the recruitment strategy in this species. Two opposed recruitment strategies have been described for partially clonal species, especially in terrestrial plants and in marine sessile organisms (seagrass and corals), the “initial seedling recruitment” (ISR) and the “repeated seedling recruitment” (RSR) (Eriksson, 1993). In ISR species, populations originate from a single event of colonization, and no local sexual recruitment occurs after the establishment of the initial cohort. On the opposite, in RSR species, new MLGs appear continuously in local populations, resulting from sexual recruitment. Partially clonal species are likely to present recruitment strategies mixing both extremes in different proportions. Thus, the ISR strategy seems to be privileged in meadows of seagrass species such as *Zostera marina* (Thorsten, Christoffer, Wytze, & Jeanine, 1999), or in some coral populations of *Pocillopora damicornis sensu lato* (Gorospe & Karl, 2013), *Pocillopora damicornis* β (Gélin et al., 2017), *Acropora palmata,* and *Acropora cervicornis* (Japaud, Bouchon, Manceau, & Fauvelot, 2015) whereas the RSR strategy seems predominant in populations of the seagrass *Cymodocea nodosa* (Ruggiero, Capone, Pirozzi, Reusch, & Procaccini, 2005).

The prevalence of few dominant MLGs that seem to be present for several years highlights the predominance of the ISR strategy (Eriksson, 1993) in *S. chloronotus* populations at Reunion Island. ISR strategy is dominant in populations with important intraspecific competition or with a low level of environmental and demographic variations, as fitter MLGs settle at the expense of others (Eriksson, 1993; Hartnett & Bazzaz, 1985). Nevertheless, the presence of rare MLGs not found two years apart shows the settlement of new MLGs, allowing the maintenance of some genetic diversity in populations under highly variable environments. This pattern showing some dominant clones with transient rare ones has already been highlighted in *Z. marina* meadows using a temporal genetic monitoring (Becheler et al., 2014). If the predominance of the ISR strategy seems congruent with the high densities and thus the potentially high intraspecific competition of *S. chloronotus* in HIGH2, it is more difficult to explain the predominance of one clone in LOW3 (Saint Pierre). At this site, *S. chloronotus* densities are very low and individuals are frequently disturbed by ocean swells and currents, freeing microsites for new recruits, more in adequacy with a RSR strategy. Some further work is required to fully understand patterns triggering asexual propagation in this population.

Our findings on the importance of clonal reproduction in the Reunion Island populations may benefit aquaculture production of *S. chloronotus*. Aquaculture of sea cucumbers is realized in different ways, in land‐based aquaculture ponds or by sea ranching: Individuals are maintained *in situ* in more or less closed areas with no additional food, the juveniles either coming from hatchery process or from the wild (for a review, see Purcell, Hair, et al., 2012; Purcell, Samyn, et al., 2012). The weak clonal propagation of *S. chloronotus* and the high fission rate in its populations could allow sea ranching with juveniles present in the wild, as soon as local conditions are optimal, especially regarding the feeding ground. Its short‐range propagation also spares the use of sea pens to close the ranching areas, diminishing the costs of pen construction, cleaning, and repairing (Robinson & Pascal, 2012). Second, we highlighted here a specific clonal structure within Reunion Island reefs, each with a specific assemblage of few clones that seem locally adapted. So it might be wise to farm the animals where they are aggregated and not to translocate them too far from their source environment. Understanding which local environmental factors drive these high rates of asexual reproduction and these high‐density populations, especially in Reunion Island, has to be addressed by future research.

## 5 CONCLUSION

To our knowledge, this study is the first to study genetic structure of fissiparous sea cucumbers at different spatial scales, adding a temporal monitoring. It reveals as follows:

- The importance of asexual reproduction to maintain populations of *S. chloronotus* and their weak clonal richness: While evidently well‐adapted to local conditions, this lack of genetic diversity may reduce their abilities to adapt to new environmental conditions.
- The high stability of clonal composition through time: The maintenance of the same predominant clones over seasons and years with weak inputs through sexual reproduction indicates the prevalence of the Initial Seedling Recruitment strategy in *S. chloronotus*. It is probably due to the stability of environmental conditions.
- A limited clonal propagation at the reef scale even if reefs seemed connected through larval dispersal, which could potentially influence the gene flow among *S. chloronotus* populations.

During this study, no exceptional events, such as cyclones, pollution, or fisheries, occurred that could have completely changed the clonal structure of these populations. In perspective, it would be interesting to pursue the temporal monitoring of these populations to assess the impact of exceptional events on their evolution in terms of clonal composition and genetic diversity.

## CONFLICT OF INTEREST

None declared.

## DATA ACCESSIBILITY

Microsatellite genotypes: Dryad doi: 10.5061/dryad.n5140.

## AUTHOR CONTRIBUTIONS

AP, PG, AB, GB, SG, PF, and HM performed sampling. AP, PG, AB, SG, GB, and HM analyzed data. PF and HM designed research. AP, PG, PF, and HM wrote the manuscript.
