# Supplementary material for: Clonal structure through space and time: High stability in the holothurian Stichopus chloronotus (Echinodermata)
Source: Ecol Evol. 2017 Aug 14;7(18):7534–47. doi: 10.1002/ece3.3285 (PMC5606904; doi:10.1002/ece3.3285)
Supplement: Supplementary file 1 [file ECE3-7-7534-s001.docx]

**Appendix S1.** Panels used to multiplex loci post-PCR and allelic ranges (in base pairs, including M13 tail)**.**

| **Locus Name** | **Panel** | **Fluorochrome** | **Allelic Range** | **Reference** |
| --- | --- | --- | --- | --- |
| Sc10 | 1 | 6-FAM | 142-164 | Taquet et al. 2011 |
| Sc09 | 1 | VIC | 200-206 | Taquet et al. 2011 |
| Sc43 | 1 | 6-FAM | 233-239 | Taquet et al. 2011 |
| Sc01 | 1 | VIC | 323-326 | Taquet et al. 2011 |
| Sc24 | 2 | 6-FAM | 148-158 | Taquet et al. 2011 |
| Sm007 | 2 | VIC | 172-176 | Xia et al. 2010 |
| Sc29 | 2 | NED | 212-218 | Taquet et al. 2011 |
| Sc33 | 2 | 6-FAM | 260-266 | Taquet et al. 2011 |
| Sm014 | 2 | VIC | 355-365 | Xia et al. 2010 |
