# Supplementary material for: Clonal structure through space and time: High stability in the holothurian Stichopus chloronotus (Echinodermata)
Source: Ecol Evol. 2017 Aug 14;7(18):7534–47. doi: 10.1002/ece3.3285 (PMC5606904; doi:10.1002/ece3.3285)
Supplement: Supplementary file 2 [file ECE3-7-7534-s002.docx]

**Appendix S2.** Simulated clonal richnesses (Dorken & Eckert 2001) under sexual reproduction.

For each site (i.e. population) at T0_cold_, were simulated the multi-locus genotypes obtained at the next generation for each population considering (1) only sexual reproduction including selfing (i.e. no fission), (2) panmixia, (3) unlinked loci and (4) constant population size. For this demonstration, was considered only T0_cold_, as no significant clonal composition changes were observed between seasons and sampling periods. The simulated multi-locus genotypes were created by randomly sampling two alleles for each of the studied loci in the gamete pool (corresponding to the allele frequencies in each observed population). For each observed population, 10^3^ datasets were simulated, and a mean clonal richness *R_sim_* was then assessed, as well as the 95% confidence interval.

| T0_cold_ | **HIGH1** | **LOW1** | **HIGH2** | **LOW2** | **HIGH3** |
| --- | --- | --- | --- | --- | --- |
| ***N_sim_*** | 128 | 32 | 128 | 32 | 128 |
| ***R_sim_*** | 0.891  [0.835-0.937] | 0.966  [0.966-0.967] | 0.273  [0.272-0.274] | 0.884  [0.881-0.887] | 0.684  [0.683-0.686] |
| ***R*** | 0.071*** | 0.133*** | 0.040*** | 0.065*** | 0.134*** |

*N_sim_* number of multi-locus genotypes simulated for each population (corresponding to the number of individuals of each observed populations); *R_sim_* mean clonal richness of the 10^3^ simulated datasets for each observed population; *R* clonal richness observed for each population (cf Table 2). In brackets is the 95% confidence interval and the probability that the observed *R* value was significantly lower than the simulated ones is also indicated (***: *P* < 0.001).
