# Supplementary material for: Clonal structure through space and time: High stability in the holothurian Stichopus chloronotus (Echinodermata)
Source: Ecol Evol. 2017 Aug 14;7(18):7534–47. doi: 10.1002/ece3.3285 (PMC5606904; doi:10.1002/ece3.3285)
Supplement: Supplementary file 3 [file ECE3-7-7534-s003.docx]

**Appendix S3.** Genetic differentiation between sites estimated with Weir and Cockerham’s *F_ST_* and Jost’s *D_est_*.

**(a)** Genetic differentiation between sites for all seasons and sampling periods estimated with Weir and Cockerham’s *F_ST_* and Jost’s *D_est_* and keeping all individuals. Values in the lower-left matrix are *F_ST_* estimates, values in the upper-right matrix are *D_est_* estimates. Numbers in parentheses are numbers of individuals kept for the analyses.

| **T0_cold_** | |  |  |  |  |
| --- | --- | --- | --- | --- | --- |
|  | HIGH1 (127) | HIGH2 (127) | HIGH3 (128) | LOW1 (31) | LOW2 (32) |
| HIGH1 | - | **0.09** | **0.08** | 0.00 | **0.13** |
| HIGH2 | **0.17** | - | **0.02** | **0.09** | **0.20** |
| HIGH3 | **0.11** | **0.06** | - | **0.08** | **0.19** |
| LOW1 | 0.00 | **0.20** | **0.11** | - | **0.13** |
| LOW2 | **0.18** | **0.37** | **0.27** | **0.18** | - |

| **T0_warm_** | |  |  |  |  |
| --- | --- | --- | --- | --- | --- |
|  | HIGH1 (126) | HIGH2 (127) | HIGH3 (128) | LOW1 (32) | LOW2 (31) |
| HIGH1 | - | **0.10** | **0.09** | **0.02** | **0.12** |
| HIGH2 | **0.19** | - | **0.02** | **0.09** | **0.19** |
| HIGH3 | **0.14** | **0.07** | - | **0.08** | **0.19** |
| LOW1 | **0.03** | **0.19** | **0.12** | - | **0.13** |
| LOW2 | **0.20** | **0.35** | **0.29** | **0.18** | - |

| **T2_cold_** | |  |  |  |  |
| --- | --- | --- | --- | --- | --- |
|  | HIGH1 (64) | HIGH2 (64) | HIGH3 (61) | LOW2 (45) | LOW3 (46) |
| HIGH1 | - | **0.07** | **0.06** | **0.11** | **0.10** |
| HIGH2 | **0.13** | - | **0.02** | **0.18** | **0.14** |
| HIGH3 | **0.09** | **0.07** | - | **0.19** | **0.17** |
| LOW2 | **0.15** | **0.31** | **0.26** | - | **0.05** |
| LOW3 | **0.20** | **0.36** | **0.33** | **0.12** | - |

| **T2_warm_** | |  |  |  |  |
| --- | --- | --- | --- | --- | --- |
|  | HIGH1 (63) | HIGH2 (64) | HIGH3 (64) | LOW2 (48) | LOW3 (48) |
| HIGH1 | - | **0.12** | **0.04** | **0.10** | **0.10** |
| HIGH2 | **0.21** | - | **0.04** | **0.17** | **0.14** |
| HIGH3 | **0.21** | **0.11** | - | **0.19** | **0.18** |
| LOW2 | **0.16** | **0.30** | **0.28** | - | **0.04** |
| LOW3 | **0.23** | **0.36** | **0.36** | **0.11** | - |

white not bold: (*P*> 0.05); bold: significant values (*P*< 0.05) with: white: *P*< 0.05; light grey: *P*< 0.01; grey: *P*< 0.001.

**(b)** Genetic differentiation between high-density sites all sampling dates pooled estimated with Weir and Cockerham’s *F_ST_* and Jost’s *D_est_* and keeping all individuals. Values in the lower-left matrix are *F_ST_* estimates, values in the upper-right matrix are *D_est_* estimates. Numbers in parentheses are numbers of individuals kept for the analyses.

|  | HIGH1 (380) | HIGH2 (382) | HIGH3 (381) |
| --- | --- | --- | --- |
| HIGH1 | **-** | **0.09** | **0.08** |
| HIGH2 | **0.17** | **-** | **0.02** |
| HIGH3 | **0.12** | **0.06** | **-** |

grey and bold: *P*< 0.001.

**(c)** Genetic differentiation between high-density sites all sampling dates pooled estimated with Weir and Cockerham’s *F_ST_* and Jost’s *D_est_* and keeping one representative per MLG. Values in the lower-left matrix are *F_ST_* estimates, values in the upper-right matrix are *D_est_* estimates. Numbers in parentheses are numbers of individuals kept for the analyses.

|  | HIGH1 (22) | HIGH2 (16) | HIGH3 (16) |
| --- | --- | --- | --- |
| HIGH1 | **-** | **0.03** | **0.02** |
| HIGH2 | **0.04** | **-** | 0.01 |
| HIGH3 | **0.04** | **0.03** | **-** |

white not bold: (*P*> 0.05); bold: significant values (*P*< 0.05) with: white: *P*< 0.05; light grey: *P*< 0.01.
