# Supplementary material for: Clonal structure through space and time: High stability in the holothurian Stichopus chloronotus (Echinodermata)
Source: Ecol Evol. 2017 Aug 14;7(18):7534–47. doi: 10.1002/ece3.3285 (PMC5606904; doi:10.1002/ece3.3285)

**Appendix S4.** Network topologies of multi-locus genotypes (MLGs) identified in *Stichopus chloronotus* individuals from the four sites common to both sampling periods: (**a**) T0 and (**b**) T2, based on Rozenfeld’s distance. Only links with distances smaller or equal to the percolation threshold (Dpe = 0.10 in T0 and Dpe = 0.08 in T2) are presented. Nodes, representing MLGs, are not arranged according to their geographic coordinates. Node size is proportional to the number of individuals harbouring each MLG. Node colours correspond to reefs.


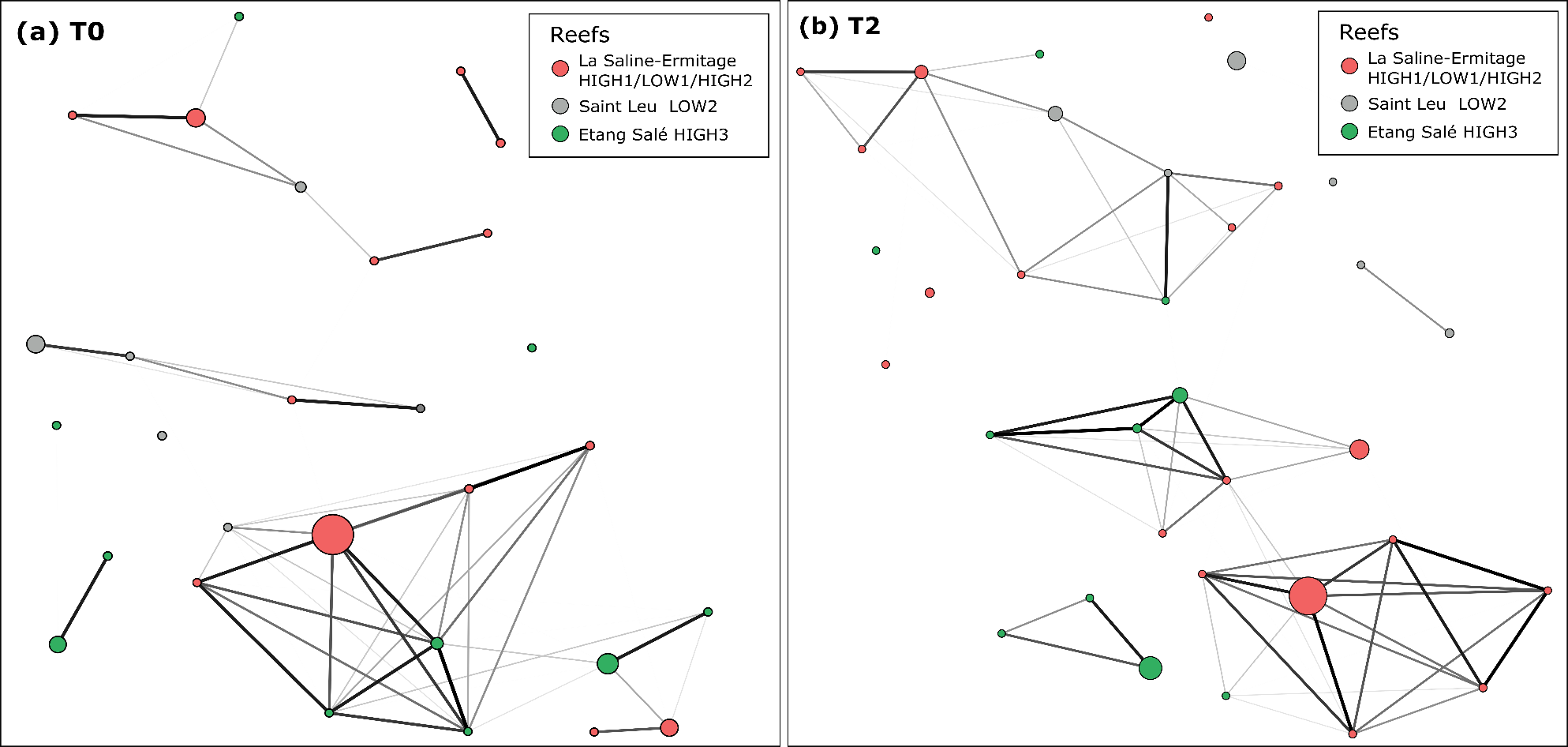

Supplement: Supplementary file 4 [file ECE3-7-7534-s004.docx]
