# Supplementary material for: Clonal structure through space and time: High stability in the holothurian Stichopus chloronotus (Echinodermata)
Source: Ecol Evol. 2017 Aug 14;7(18):7534–47. doi: 10.1002/ece3.3285 (PMC5606904; doi:10.1002/ece3.3285)
Supplement: Supplementary file 5 [file ECE3-7-7534-s005.docx]

**Appendix S5.** Clustering coefficients of the networks constructed at the station level

| **Site** | **Station** | **T0_cold_** | **T0_warm_** | **T2_cold_** | **T2_warm_** |
| --- | --- | --- | --- | --- | --- |
| **HIGH1** | S1 | 0.29 | 0.80 | - | - |
|  | S2 | 0.74 | 0.83 | 0.45 | 0.31 |
| **LOW1** |  | 0.00 | 0.28 | - | - |
| **HIGH2** | S1 | 0.00 | 0.58 | 0.00 | 0.39 |
|  | S2 | 0.77 | 0.68 | - | - |
| **LOW2** |  | 0.00 | 0.47 | 0.00 | 0.00 |
| **HIGH3** | S1 | 0.67 | 0.50 | 0.44 | 0.47 |
|  | S2 | 0.78 | 0.33 | - | - |
| **LOW3** |  | - | - | - | - |
