# Supplementary material for: Clonal structure through space and time: High stability in the holothurian Stichopus chloronotus (Echinodermata)
Source: Ecol Evol. 2017 Aug 14;7(18):7534–47. doi: 10.1002/ece3.3285 (PMC5606904; doi:10.1002/ece3.3285)

**Appendix S6.** Statistical analyses concerning population density and individual size in *Stichopus chloronotus* in Reunion Island.

**(a)** *Stichopus chloronotus* mean population densities of the high-density sites (HIGH1/HIGH2/HIGH3) at the sampling period T2 according to season (cold/warm).


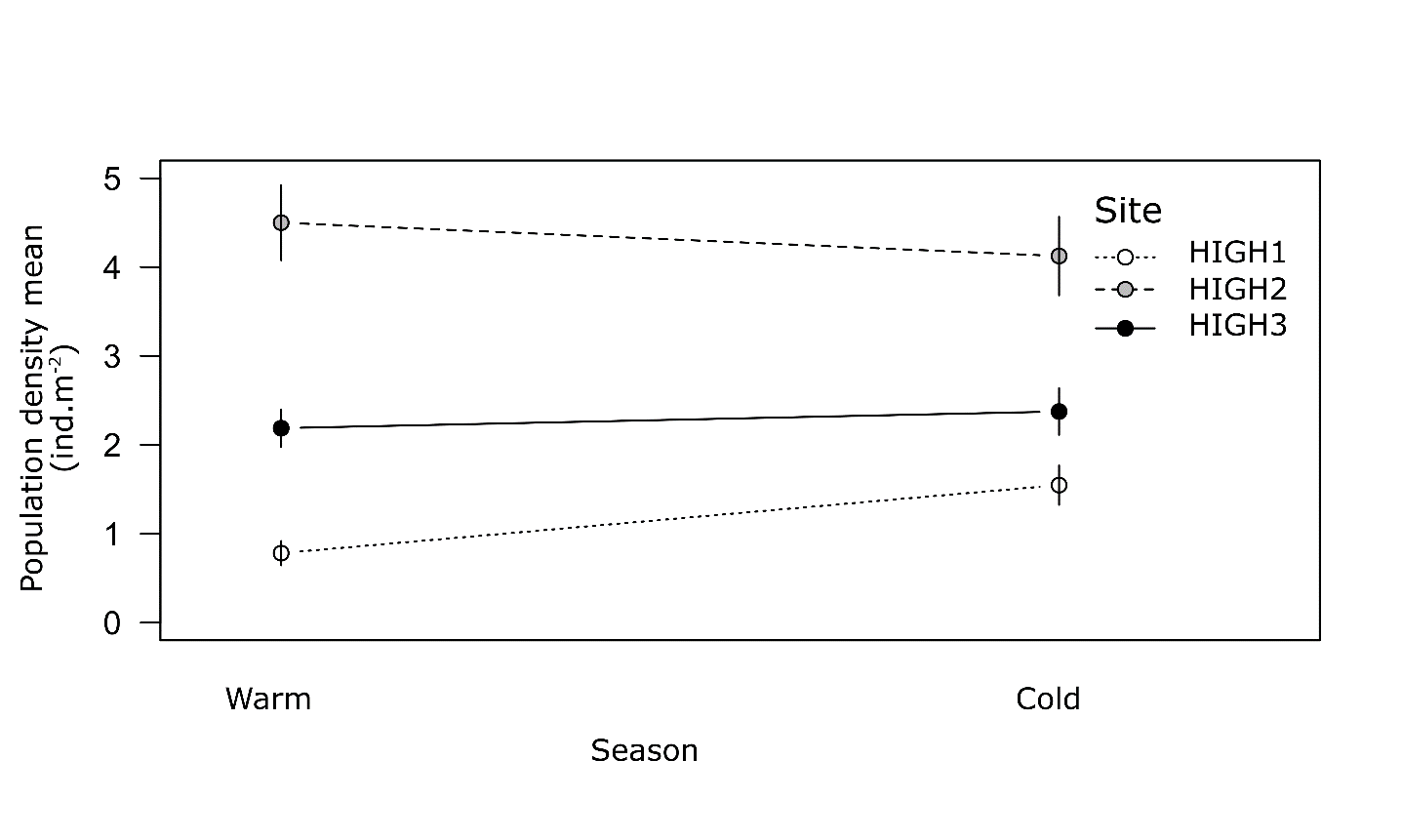


**(b)** Table of the two-way ANOVA performed to test the influence of season and site on population density in *Stichopus chloronotus*, for high density sites HIGH1, HIGH2 and HIGH3 at T2; *: *P* < 0.05;**: *P* < 0.01; ***: *P* < 0.001.

| **T2** | **Df** | **Sum Sq** | **Mean Sq** | **F value** | ***P*** |
| --- | --- | --- | --- | --- | --- |
| **Season (cold or warm)** | 1 | 3.57 | 3.57 | 0.60 | 4.38x10^-1^ |
| **Site (HIGH1, HIGH2 or HIGH3)** | 2 | 652.23 | 326.12 | 55.12 | <2x10^-16^ *** |
| **Interaction Season-Site** | 2 | 20.82 | 10.41 | 1.76 | 1.74x10^-1^ |
| **Residuals** | 378 | 2236.55 | 5.92 |  |  |

**(c)** Mean population density (ind.m^-2^) of *Stichopus chloronotus* of high-density sites sampled at T2. Vertical bars are for standard errors. Letters a, b, c, d, indicate significant variations of sizes between sites (pairwise-t-tests with FDR correction).


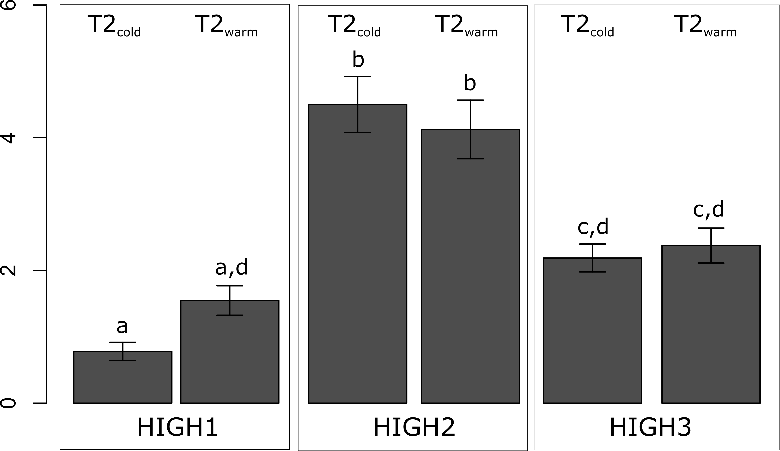


**(d)** Mean size of *Stichopus chloronotus* individuals sampled in Reunion Island according to season (cold/warm) and density (low/high).

**
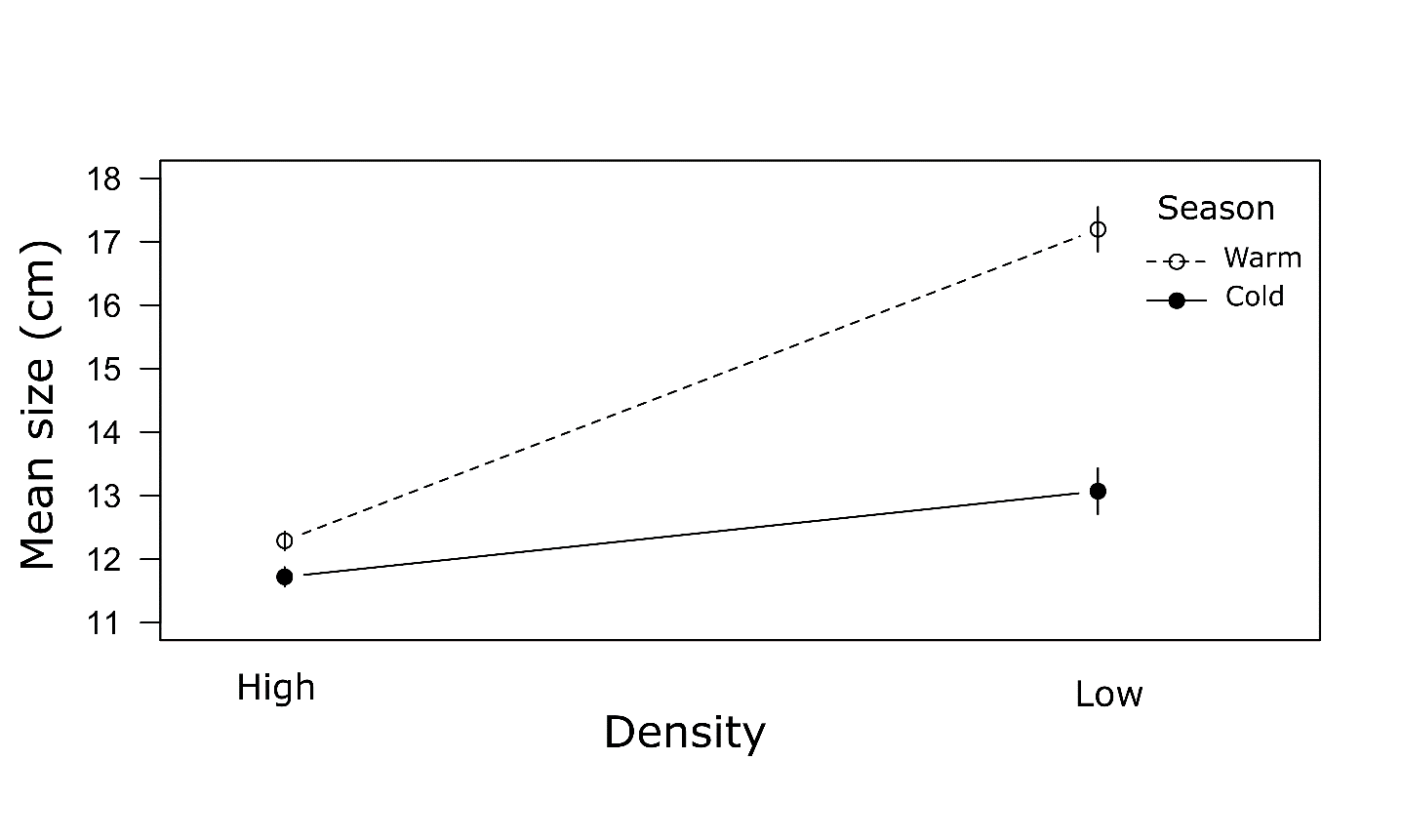
**

**(e)** Table of the two-way ANOVA performed to test the influence of season and density on *Stichopus chloronotus* individual sizes; *: *P* < 0.05;**: *P* < 0.01; ***: *P* < 0.001.

|  | **Df** | **Sum Sq** | **Mean Sq** | **F value** | ***P*** |
| --- | --- | --- | --- | --- | --- |
| **Season (cold or warm)** | 1 | 2439.4 | 2439.37 | 167.539 | <2.2x10^-16^ *** |
| **Density (high or low)** | 1 | 657.9 | 657.87 | 45.184 | 2.56x10^-11^ *** |
| **Interaction Season-Density** | 1 | 788.1 | 788.08 | 54.126 | 3.12x10^-13^ *** |
| **Residuals** | 1466 | 21345.1 | 14.56 |  |  |

**(f)** Mean sizes (cm) of *Stichopus chloronotus* individuals sampled at T0 and T2 at all sites and all seasons. Vertical bars are for standard errors. Letters a, b, c, d, e, indicate significant variations of sizes between sites estimated for T0 and T2 independently (pairwise-t-tests with FDR correction). Light grey indicates low-density sites and dark grey indicates high-density sites.


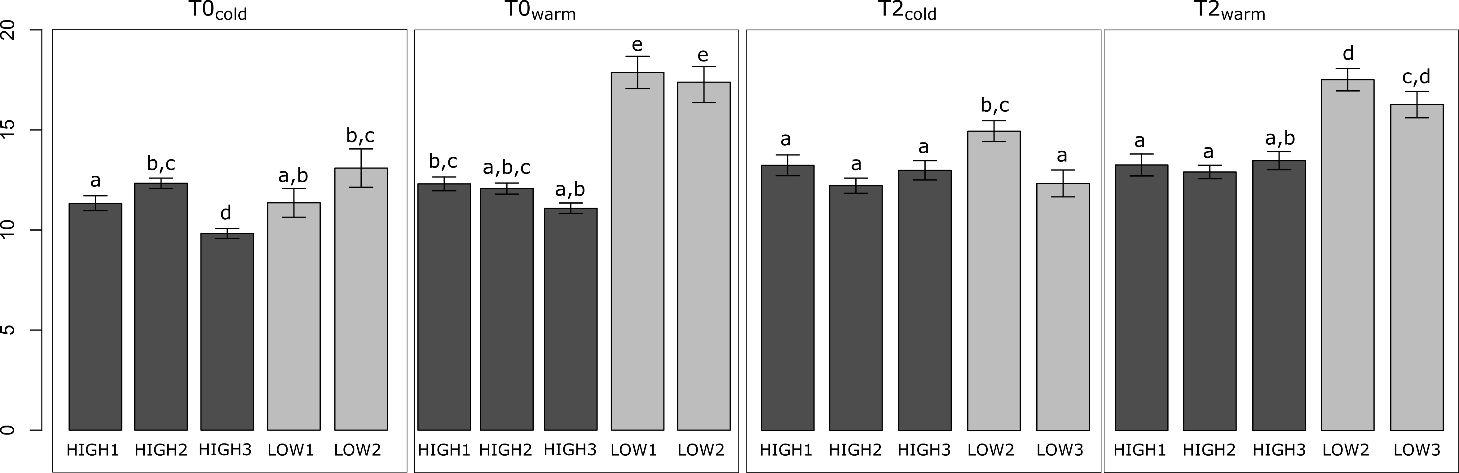

Supplement: Supplementary file 6 [file ECE3-7-7534-s006.docx]
